# Maternal exposure to a high-magnitude earthquake during pregnancy influences pre-reading skills in early childhood

**DOI:** 10.1038/s41598-021-88767-7

**Published:** 2021-04-29

**Authors:** Luis Federico Bátiz, Yasna K. Palmeiro-Silva, Gregory E. Rice, Lara J. Monteiro, Albert M. Galaburda, Roberto Romero, Mahesh A. Choolani, Ursula Wyneken, Pelusa Orellana, Sebastián E. Illanes

**Affiliations:** 1 School of Medicine, Faculty of Medicine, Universidad de los Andes, Santiago, Chile; 2 Program in Neuroscience, Centro de Investigación e Innovación Biomédica (CiiB), Universidad de los Andes, Santiago, Chile; 3 Institute for Global Health, University College London, London, UK; 4 School of Nursing, Universidad de los Andes, Santiago, Chile; 5 Centre for Clinical Research, University of Queensland, Brisbane, QLD Australia; 6 Program in Biology of Reproduction, Centro de Investigación e Innovación Biomédica (CiiB), Universidad de los Andes, Santiago, Chile; 7 Department of Neurology, Harvard Medical School, Boston, MA USA; 8 Perinatology Research Branch, Eunice Kennedy Shriver National Institute of Child Health and Development, National Institutes of Health, Department of Health and Human Services, Bethesda, MD 20892 USA; 9 Perinatology Research Branch, Eunice Kennedy Shriver National Institute of Child Health and Development, National Institutes of Health, Department of Health and Human Services, Detroit, MI 48201 USA; 10 Department of Obstetrics and Gynecology, University of Michigan, Ann Arbor, MI 48109 USA; 11 Department of Epidemiology and Biostatistics, Michigan State University, East Lansing, MI 48824 USA; 12 Center for Molecular Obstetrics and Genetics, Wayne State University, Detroit, MI 48201 USA; 13 Detroit Medical Center, Detroit, MI 48201 USA; 14 Department of Obstetrics and Gynecology, Florida International University, Miami, FL 33199 USA; 15 Division of Maternal Fetal Medicine, Department of Obstetrics and Gynecology, Yong Loo Lin School of Medicine, National University of Singapore, Singapore, Singapore; 16 School of Education, Universidad de los Andes, Santiago, Chile; 17 Department of Obstetrics and Gynecology, Faculty of Medicine, Universidad de los Andes, Santiago, Chile

**Keywords:** Intrauterine growth, Epigenetics and plasticity, Stress and resilience, Reading

## Abstract

Exposure to an adverse prenatal environment can influence fetal development and result in long-lasting changes in the offspring. However, the association between maternal exposure to stressful events during pregnancy and the achievement of pre-reading skills in the offspring is unknown. Here we examined the association between prenatal exposure to the Chilean high-magnitude earthquake that occurred on February 27th, 2010 and the development of early reading precursors skills (listening comprehension, print knowledge, alphabet knowledge, vocabulary, and phonological awareness) in children at kindergarten age. This multilevel retrospective cohort study including 3280 children, of whom 2415 were unexposed and 865 were prenatally exposed to the earthquake shows substantial evidence that maternal exposure to an unambiguously stressful event resulted in impaired pre-reading skills and that a higher detrimental effect was observed in those children who had been exposed to the earthquake during the first trimester of gestation. In addition, females were more significantly affected by the exposure to the earthquake than their male peers in alphabet knowledge; contrarily, males were more affected than females in print knowledge skills. These findings suggest that early intervention programs for pregnant women and/or children exposed to prenatal stress may be effective strategies to overcome impaired pre-reading skills in children.

## Introduction

Consistent with the fetal programming hypothesis articulated by Barker in the 1990s^1,2^, a growing body of evidence indicates that exposure to an adverse prenatal environment can influence fetal development, resulting in long-lasting effects on the offspring^3–7^. Prenatal stress has been reported to affect fetal growth^8^, as well as childhood behavior and cognitive performance^8–11^. In fact, different types of stressors, e.g., exposure to adverse life events or exposure to natural and human-made hazards such as earthquakes, hurricanes, ice storms, or terrorist acts during pregnancy, have been linked to child behavioral problems^12,13^, deficient cognitive abilities and fearfulness in infancy^14–17^, and higher risks for neurodevelopmental disorders such as schizophrenia^18,19^, autism spectrum disorder (ASD) and attention deficit hyperactivity disorder (ADHD)^20,21^. Furthermore, structural, and functional studies have demonstrated changes in various regions of children’s brains exposed to prenatal maternal stress^22–26^.

Effects of prenatal stress likely depend upon complex interactions between fetal genetic backgrounds, fetal sex, and gestational age at the time of exposure^7,27^. Some studies have identified sexually dimorphic responses to prenatal stress^28,29^, and others have demonstrated that the timing of exposure, e.g., early, mid-, or late gestation, may play a critical role in determining neurodevelopmental outcomes^30,31^. Oyarzo et al. and Palmeiro-Silva et al. reported that exposure to a high-intensity earthquake during pregnancy was linked to adverse prenatal outcomes, such as early delivery and reduced offspring length and head circumference, depending on fetal sex and trimester of exposure^8,32^.

Whereas previous studies have demonstrated that prenatal stress is associated with poorer performance at school in their offspring^17,33,34^, only few have examined the link between prenatal exposure to stressful events and the development of reading precursors at kindergarten stage^10^ and more advanced levels. Literacy achievement, i.e., the quantitative assessment of ability to read and write, is an outcome measure of language development^35,36^ and depends on domain-general cognitive processes^37–39^. Children with language impairment have a lower literacy achievement and higher rates of reading disorders^35,36,40–42^. Thus, the development of reading precursors and the acquisition of enabling skills for reading, such as listening comprehension, vocabulary knowledge, and phonological awareness^43^ during early childhood ages, could be considered as a significant outcome measure of cognitive and language development, and could predict reading competence in later life^33,40,44,45^.

This study examined the association between prenatal exposure to the earthquake that occurred in Chile on February 27th, 2010 (known as Chilean 27F earthquake) and the performance of kindergarten children on five pre-reading skills: listening comprehension, print knowledge, alphabet knowledge, vocabulary, and phonological awareness.

## Results

In this study, 3280 children were studied, of whom 2415 (73.63%) were considered unexposed and 865 (26.37%) exposed to the earthquake. The distribution by sex (male vs female) was approximately 50% in unexposed and exposed children across all cohorts (Table 1). Considering exposed children, it was estimated that the majority was exposed during the second trimester; however, in general, the distribution was approximately uniform across all trimesters (Table 2). Kindergarten students were assessed at the beginning of each academic year for three consecutive years, using the DIALECT platform, a validated Spanish reading diagnostic instrument^47,48^. The skills (i) listening comprehension, (ii) alphabet knowledge, (iii) print knowledge, (iv) phonological awareness, and (v) vocabulary were measured considering four achievement categories according to previous reports^49^ (Table 3).

**Table 1:** Distribution of unexposed and exposed children by sex and year of study.

Year	Unexposed (n = 2415)			Exposed (n = 865)			Total
	Male	Female	Total	Male	Female	Total	
2015	342 (50.82%)	331 (49.18%)	673	33 (45.83%)	39 (54.17%)	72	745
2016	176 (45.60%)	210 (54.40%)	386	389 (49.55%)	396 (50.45%)	785	1171
2017	632 (46.61%)	724 (53.39%)	1356	4 (50.00%)	4 (50.00%)	8	1364
Total	1150	1265	2415	426	439	865	3280

Data are presented as n and (%).

**Table 2:** Distribution of exposed children by sex and timing of exposure.

	Exposed (n = 865)			Total
	First trimester	Second trimester	Third trimester	
Male	146 (34.27%)	163 (38.26%)	117 (27.47%)	426
Female	146 (33.26%)	174 (39.63%)	119 (27.11%)	439
Total	292	337	236	865

**Table 3:** Pre-Reading skills assessed by DIALECT platform.

Reading precursors	Tasks	Maximum score (points)	Categories of achievement
Listening comprehension	Students listen to a brief story and respond to 5 questions (two testing literal comprehension, two testing inferential comprehension, and one vocabulary question) by clicking on the picture that best represents the answer	5 points	1: Delayed: 0–1 pt 2: Normal: 2–3 pts 3: Very good: 4 pts 4: Outstanding: 5 pts
Alphabet knowledge	Students identify a letter they heard from a set of 3 possible letters	27 points	1: Delayed: < 9 pts 2: Normal: 9–16 pts 3: Very good: 17–25 pts 4: Outstanding: > 25 pts
Print knowledge	Students are asked to identify (i) various book parts, such as title and author, and (ii) text components, such as word, upper-case/lower-case letters, and some punctuation marks	10 points	1: Delayed: 0–3 pts 2: Normal: 4–7 pts 3: Very good: 8–9 pts 4: Outstanding: 10 pts
Phonological awareness	There are several phonological awareness tasks; in this case, two phonological tasks were administered. (A) Students listen to a set of sounds and are asked to process them together and blend phonemes to form a word that corresponded to one of three pictures depicted on a screen. (B) Students are asked to identify pictures representing words that had the same initial sound	(A) 5 points (B) 5 points Total: Mean (A:B); 5 points	1: Delayed: 0–2 pts 2: Normal: 3 pts 3: Very good: 4 pts 4: Outstanding: 5 pts
Vocabulary	Students listen to a target word and select one of four screen icons that corresponded to the word	81 points (t-score)	1: Delayed: < 44 pts 2: Normal: 45–51 pts 3: Very good: 52–56 pts 4: Outstanding: > 56 pts

**Figure 1:**
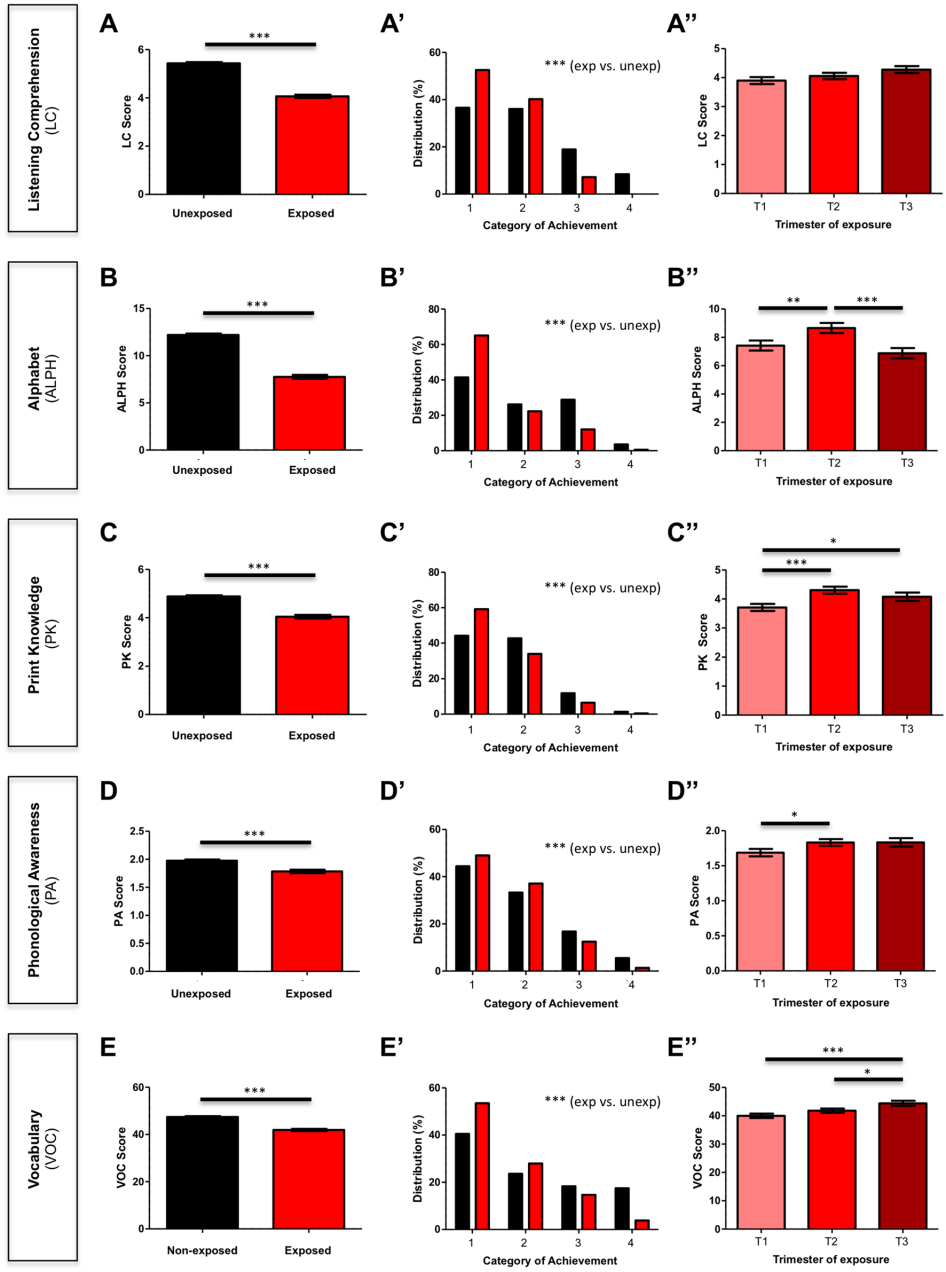
Effect of prenatal exposure and timing of exposure to 27F earthquake on pre-reading skills. (**A**–**E**) Scores obtained by unexposed and exposed children for each pre-reading skill. ***p < 0.001 (Mann–Whitney *U* test). (**A**′–**E**′) Distribution (%) of children per category of achievement. Categories considered were 1: “delayed”; 2: “normal”; 3: “very good”; 4: “outstanding. ***p < 0.001 exposed vs. unexposed (Fisher’s exact test). (**A**″–**E**″) Scores obtained by exposed children according to the gestational trimester of exposure. T1: first trimester; T2: second trimester; T3: third trimester. *p < 0.05; **p < 0.01; ***p < 0.001 (Dunn’s test—multiple pairwise comparisons).

Across all variables of interest, children exposed to the earthquake scored lower than their unexposed peers (p < 0.0001) (Fig. 1A–E). Significant differences between exposed and unexposed children were found for listening comprehension, where the mean score of exposed children was 25% less than unexposed ones [mean ± SEM: 5.431 ± 0.050 (unexposed) vs. 4.063 ± 0.068 (exposed)], and alphabet knowledge, where the mean score of exposed children was 36% less than unexposed ones [mean ± SEM: 12.192 ± 0.154 (unexposed) vs. 7.758 ± 0.210 (exposed)] (Fig. 1A,B).

When comparing children’s performance on different categories of achievement, we observed an overrepresentation of all children in the lower achievement categories (categories 1 and 2) in both groups (40% on average) (Fig. 1A′–E′); however, the percentage of exposed children in lower achievement categories (43.9% on average) was significantly greater than that of unexposed children (36.9% on average). On the other hand, the percentage of children who scored at higher levels (categories 3 and 4) was also higher among the unexposed group (13.1% vs 5.9%) (Fig. 1A′–E′).

When analyzing children’s scores according to the gestational trimester in which they were exposed to the earthquake, we observed that the scores of exposed children at any of the three trimesters were lower than the scores of unexposed children (p < 0.05). In addition, children who were exposed during the first trimester had lower scores than those exposed in the second or third trimester in four out of the five analyzed variables. On the other hand, the second and third trimesters were not equivalent; children that had been exposed in the third trimester scored lower than those exposed in the second trimester in alphabet knowledge (Fig. 1B″).

**Figure 2:**
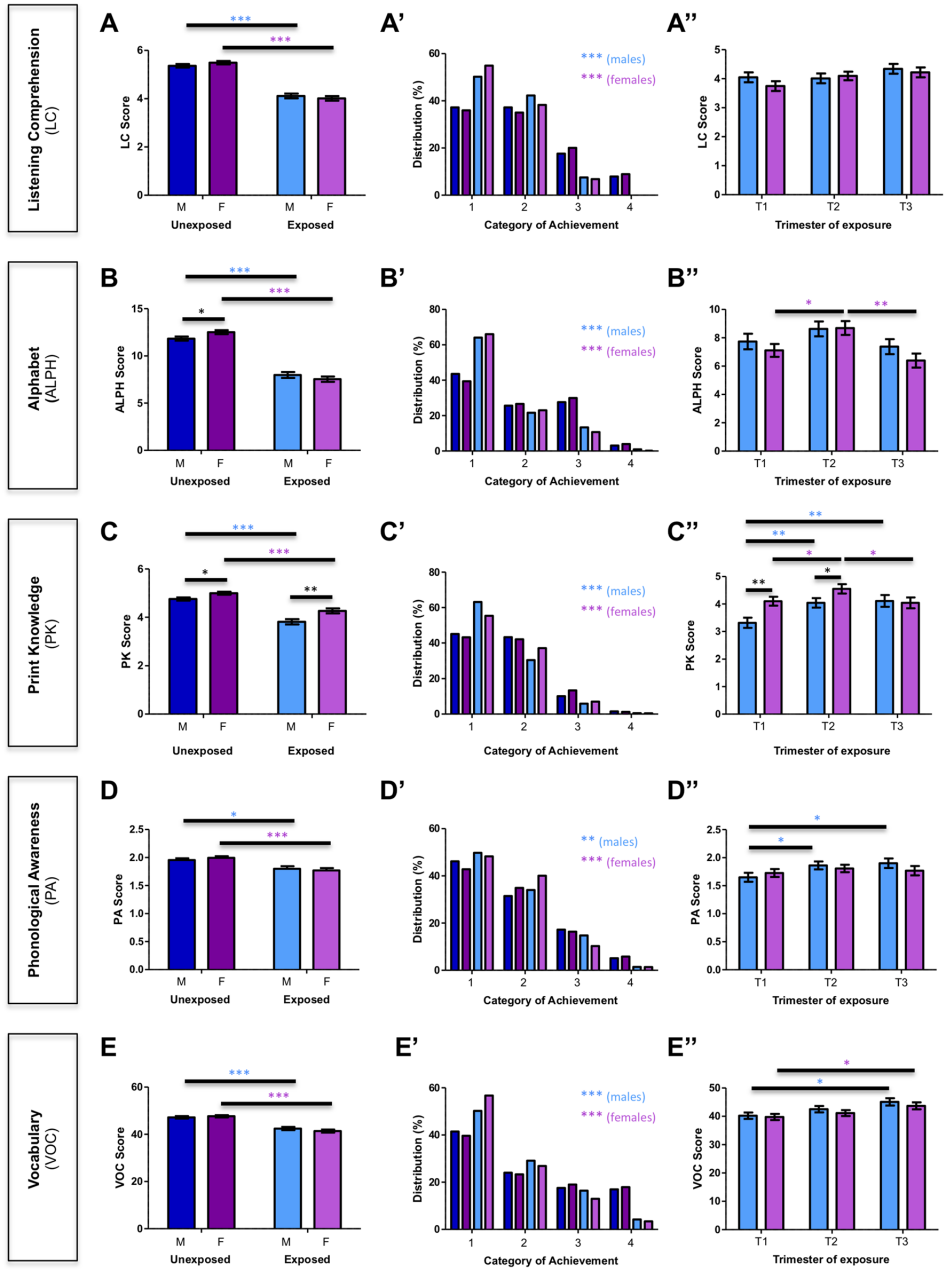
Sex differences of prenatal exposure and timing of exposure to 27F earthquake on pre-reading skills. (**A**–**E**) Scores obtained by unexposed and exposed children by sex. *M* male, *F* female. *p < 0.05; **p < 0.01; ***p < 0.001 (Mann–Whitney *U* test). (**A**′–**E**′) Distribution (%) of females and males per category of achievement. Categories considered were 1: “delayed”; 2: “normal”; 3: “very good”; 4: “outstanding”. **p < 0.01; ***p < 0.001 exposed vs. unexposed (males and females) (Fisher’s exact test). (**A**″–**E**″) Scores obtained by exposed females and males according to the gestational trimester of exposure. T1: first trimester; T2: second trimester; T3: third trimester. *p < 0.05; **p < 0.01; ***p < 0.001 (males vs. females in each trimester: Mann–Whitney *U* test; males and females comparing T1, T2 and T3: Dunn’s test—multiple pairwise comparisons).

We also compared students’ scores *vis à vis* sex. Figure 2 shows results by pre-reading skills, with groups organized by exposure and sex. Overall, we observed that both exposed females and males had lower scores than unexposed children in all analyzed variables (Fig. 2A–E). Moreover, our results showed that amongst unexposed children, males had worse performance than females in alphabet knowledge, with 0.7 points on average of difference (mean score for females = 12.5; males = 11.8; p = 0.02) and in print knowledge, with 0.3 points on average (mean score for females = 5.0; males = 4.7; p = 0.02) (Fig. 2B,C). Interestingly, in print knowledge scores, this effect appeared to be exacerbated in exposed children, increasing the magnitude of difference between females and males from 0.3 to 0.5 points (mean score for females = 4.3; males = 3.8; p = 0.002) (Fig. 2C). These results suggest that males appeared to be more affected than females in print knowledge (Fig. 2C,C′). Conversely, in alphabet knowledge, the differences observed between males and females in unexposed children (0.7 points on average higher in females) were not present in exposed children (mean score for males = 7.9; females = 7.5; p = 0.46), indicating that, in this reading skill, females are more affected by the exposure to the earthquake than their male peers. Interestingly, these differences were particularly evident when children were exposed in the first and second trimesters (Fig. 2C″).

When the scores were analyzed according to categories of achievement (Fig. 2A′–E′), we observed that a greater proportion of exposed children scored in the lower categories of achievement, particularly in category 1 (“delayed”), compared with those unexposed females and males. As expected, the proportion of exposed males and females at the other end of the distribution*, *i.e., categories 3 (“very good”) and 4 (“outstanding”), was small, with no significant differences between sexes (Fig. 2A′–E′). Even though there were no differences in the mean score achieved by exposed females and males in listening comprehension, alphabet knowledge, phonological awareness, and vocabulary (Fig. 2A,B,D,E), we observed that a higher percentage of exposed females than exposed males belonged to category 1 (“delayed”) in listening comprehension and vocabulary (Fig. 2A′,E′). On the other hand, there were more exposed males than females in the lowest category (1 or “delayed”) for print knowledge (Fig. 2C′). We did not observe significant differences between exposed males and females in the lowest category in alphabet knowledge nor in phonological awareness (Fig. 2B′,D′).

When looking at results by sex and trimester of exposure, males who were exposed to the earthquake in the first trimester of gestation had lower scores in three out of the five evaluated reading skills (print knowledge, phonological awareness and vocabulary knowledge), compared with males exposed in the second and/or third trimesters (Fig. 2C″–E″). Similarly, females exposed in the first trimester showed lower scores for alphabet knowledge, print knowledge, and vocabulary than females who were exposed in the second and/or third trimesters (Fig. 2B″,C″,E″). We only observed significant differences between females and males for print knowledge in trimesters 1 and 2, where males had lower scores than females (Fig. 2C″). Interestingly, females exposed in the third trimester showed lower scores than females exposed in the second trimester for alphabet and print knowledge, and this was not observed in males (Fig. 2B″,C″). For listening comprehension, no differences were observed among females or males across trimesters (Fig. 2A″).

Multilevel models showed consistent results when adjusting for children’s sex and multilevel structure. Table 4 summarizes the adjusted odds ratios, p-values, and 95% confidence intervals (95% CI) for each reading skill considering exposure and trimester of exposure. In general, we observed that for exposed children, the odds of achieving outstanding category (category 4) versus all the rest combined (categories 1–3) were lower than for unexposed children in all reading skills, holding all variables constant. For example, exposed children had 71% (95% CI 66–76%, p = < 0.001) less odds to achieve the outstanding category versus categories 1–3 combined in comparison to unexposed children when assessing alphabet knowledge. Regarding sex, females seemed to have higher odds to achieve the outstanding category; however, in most of the reading skills, this effect was not significant. Similarly, children who were exposed during the first, second, and third trimester had lower odds to achieve the outstanding category in comparison to unexposed children, adjusting for sex and multilevel data structure. The models revealed that exposed children during the first trimester had lower ORs than those exposed during the second or the third trimester for every reading skill. For example, when assessing listening comprehension, for children who were exposed in the first trimester the odds of outstanding category versus category 1–3 were 0.4 times lower than unexposed children (95% CI 0.31–0.5), but these ORs increase up to 0.44 (95% CI 0.35–0.54) and 0.47 (95% CI 0.37–0.61) for children who were exposed during the second and third trimester, respectively.

**Table 4:** Multilevel analysis of achievement the highest category in exposed and unexposed children.

Reading precursors	Multilevel adjusted OR	95% CI	p-value	Multilevel adjusted OR	95% CI	p-value
**Listening comprehension**						
Exposed	0.43	0.37–0.50	< 0.001			
Female	1.06	0.92–1.22	0.389			
Exposed 1 T				0.40	0.31–0.50	< 0.001
Exposed 2 T				0.44	0.35–0.54	< 0.001
Exposed 3 T				0.47	0.37–0.61	< 0.001
Female				1.06	0.92–1.23	0.386
**Print knowledge**						
Exposed	0.54	0.46–0.63	< 0.001			
Female	1.25	1.08–1.44	0.003			
Exposed 1 T				0.39	0.30–0.50	< 0.001
Exposed 2 T				0.68	0.55–0.86	0.001
Exposed 3 T				0.55	0.42–0.72	< 0.001
Female				1.25	1.08–1.45	0.003
**Alphabet**						
Exposed	0.29	0.24–0.34	< 0.001			
Female	1.16	1.00–1.35	0.045			
Exposed 1 T				0.27	0.19–0.32	< 0.001
Exposed 2 T				0.37	0.29–0.46	< 0.001
Exposed 3 T				0.24	0.18–0.33	< 0.001
Female				1.16	1.00–1.34	0.046
**Print awareness**						
Exposed	0.74	0.63–0.85	< 0.001			
Female	1.09	0.94–1.25	0.253			
Exposed 1 T				0.64	0.51–0.81	< 0.001
Exposed 2 T				0.80	0.64–0.99	0.038
Exposed 3 T				0.77	0.60–0.99	0.046
Female				1.09	0.94–1.25	0.243
**Vocabulary**						
Exposed	0.57	0.50–0.65	< 0.001			
Female	1.03	0.90–1.17	0.709			
Exposed 1 T				0.49	0.40–0.61	< 0.001
Exposed 2 T				0.57	0.46–0.69	< 0.001
Exposed 3 T				0.68	0.54–0.86	0.001
Female				1.03	0.90–1.17	0.707

T1: exposure in the first trimester of gestation; T2: exposure in the second trimester of gestation; T3: exposure in the third trimester of gestation.

## Discussion

Overall, our data showed that maternal exposure to a high-intensity earthquake during pregnancy is associated with lower reading skills scores in the offspring. Interestingly, timing of exposure was a significant factor in establishing the effect in all pre-reading skills, and sex appeared to be a factor in modifying the effect on alphabet and print knowledge. These results help to expand our understanding of the potential negative impacts that an adverse prenatal environment can have on children development. Furthermore, since these effects can be observed at early childhood ages, our results might suggest that early and focused intervention programs are needed in order to mitigate some of the negative consequences among the affected population.

This study complements the evidence on existing natural hazards’ effects on children development. The 2010 earthquake in Chile represented an event that was very likely to cause stress in pregnant people, although this relation was not evaluated directly. Most studies of prenatal stress use standardized self-reports to measure maternal psychological distress. However, other studies, such as the present study, use a gestational exposure to a stressful event to evaluate larger populations and to unravel the effects of an exposure from maternal subjective distress (recently reviewed in^25^). In this context, our study was based on a relatively large number of participants with data on exposure to the earthquake during prenatal life.

Available evidence indicates that the timely identification of reading difficulties or factors that can explain/predict them, along with adequate intervention programs, significantly improves reading and comprehension abilities^50,51^. Research in reading diagnosis and intervention has highlighted the role of these “pre-reading skills” that are strongly related to reading outcomes, and that can be assessed at kindergarten stage, before or at the beginning of reading instruction.

Previous research has associated maternal stress during pregnancy with reduced academic performance in offsprings^17,33,34^. One of these studies reported a significant correlation between prenatal maternal stress and lower marks on literacy, numeracy, and music at six years old which takes place after their first year of grammar school^34^. More recently, Aizer et al. (2016) found that in-utero stress exposure (based on comparisons of cortisol concentrations between siblings) had a significant, negative impact on verbal IQ scores and school attainment at 7 years old^17^. Similarly, Li et al*.* (2013) found that maternal antenatal exposure to several maternal life stress events was associated with changes in reading scores at the age of ten^33^. In contrast to these previous studies, which are based on achievements at school stages, we measured the acquisition of early pre-reading skills at early childhood ages. In line with this, Laplante et al*.* (2008) showed that children exposed in-utero to high levels of maternal stress, i.e., an ice storm, had lower cognitive functioning and language abilities at age 5.5 years compared to controls, even after controlling for potential pre- and postnatal confounding variables^10^. Our results are consistent with those of Laplante et al*.,* as we observed poorer vocabulary achievement in children exposed prenatally to the 27F earthquake. Additionally, we found that exposed children not only had reduced achievement scores in vocabulary development but also in other reading skills such as listening comprehension, print knowledge, alphabet knowledge, and phonological awareness.

The data obtained in this study suggest that the timing of exposure is an important factor in determining the negative impact of prenatal stress on reading skills. This is in agreement with previous findings made by Glynn et al. (2001), suggesting that during pregnancy, women become increasingly resistant to the adverse effects of stress, so early stress would have more profound effects than later stress^52^. Torche (2018) reported that exposure to an earthquake during the first trimester of pregnancy, but not during the second or third trimester, is associated with lower cognitive ability at age of 7^16^. In contrast, Li et al. (2015) reported that exposure to an earthquake in the middle and late stages of gestation, but not in the early stages, is associated with impaired visuospatial memory^53^. Here, we observed that children exposed to the earthquake in the first trimester of gestation had significantly more detrimental effects than those exposed in the second and/or third trimester.

Since the brain undergoes complex structural and organizational changes during in-utero development, prenatal insults affecting the developing brain may cause lesions or defects, which patterns depend on the stage of brain development^54^. During the first trimester, cortical neurogenic processes take place, characterized by proliferation/differentiation of neural stem/progenitor cells, and migration of newborn neurons. During the second trimester, neurogenesis continues and processes such as neuronal organization start. Finally, the third trimester is characterized by a profuse maturation and organization of the already generated structures^55^. Neuronal plasticity (i.e., the capacity of the brain tissue to compensate or reorganize after early lesions in these developmental stages) depends on the pool of cells already developed at the moment of the insult. Accordingly, if the exposure to an insult (stressful events) occurred earlier (e.g., in the first trimester), it would have a more negative effect on the compensatory potential of the brain tissue than if the exposure occurred later (e.g., during the third trimester).

The mechanisms underlying prenatal stress-induced neurodevelopmental changes in offspring, i.e., how maternal stress is transferred to the fetus and what are the fetal targets of these stress signals, remain to be fully elucidated. Studies in animal models suggest that increased transfer of maternal cortisol across the placenta to the fetus is a significant mediator of prenatal stress^15,56^. Other substances such as catecholamines, reactive oxygen species, cytokines, and/or serotonin, released under stress conditions, may mediate materno-fetal stress-transfer^57^. Interestingly, maternal stress signals can potentially modify fetal physiology by crossing the placenta and acting directly on the fetus, or by modifying placental physiology and thus secondarily acting on the fetus.

Consistently with other reports^28^, we found that prenatal exposure to the 27F earthquake had sexually dimorphic consequences in the offspring. Furthermore, these sex-related changes appeared to be linked with the timing of exposure. It is likely that differential developmental trajectories of male and female fetuses influence differential vulnerability to prenatal stress and neurodevelopmental outcomes; however, the precise mechanisms underlying sex-specific responses to prenatal stress are poorly understood. Some authors consider that the sex of the fetus may “interact” with the maternal hypothalamic–pituitary–adrenocortical (HPA) axis and contribute to sex specific consequences of early adversity^28,58,59^. On the other hand, the placenta, known to mediate or moderate some of the consequences of maternal stress for the fetus^60,61^, produces sexually dimorphic responses to intrauterine stress exposure, in particular changes in gene expression and metabolism, and can mediate sex specific programming of the fetus^62,63^.

Interestingly, Li et al*.* (2013) found that maternal antenatal exposure to several maternal life stress events was associated with lower reading scores at the age of ten years only in females^33^. Conversely, exposed males showed better scores on reading and mathematics tasks than unexposed males, suggesting that prenatal stress has differing effects on the school performance of male and female offspring^33^. As proposed by Davis and Pfaff^28^, our results suggest that it is not that females or males are more susceptible to prenatal stress, but rather that gestational exposure to stress has sexually dimorphic consequences, and factors such as timing of exposure may play a critical role in determining the sex-specific outcomes^30,31^.

This study has some limitations. First, as we analyzed secondary data, we did not have access to other important factors and postnatal influences that could affect pre-reading skills, such as educational factors, parental background and socioeconomic variables, perinatal comorbidities, and other potential modifiers or confounders. However, as the sample was purposively selected to be homogeneous, we assumed a similar distribution of these variables across all schools, cohorts, and children. Furthermore, potential temporal factors (i.e., educational or social changes pre/post earthquake) could have important effects when measuring the outcome. Nevertheless, a report by Berthelon et al. (2018) found that the 27F earthquake and its aftermath (destruction, loss of human lives) did not modify several socio-economic variables between 2009 (pre-earthquake) and 2010 (post-earthquake), including percentage of people married, years of education, percentage of people working, self-reported health, housing global quality, and mean household income; thus, concluding that socio-economic variables did not suffer relevant changes after the earthquake^65^.

Additionally, identification of causal effects of in-utero conditions on future outcomes is challenging because of the multivariate nature of the phenomena. Further studies are needed to assess the impact of school interventions’ protocols and strategies for the development of reading skills in children exposed to prenatal stressful events, such as the 27F earthquake. It is worth mentioning that we did not have information regarding the geographical location of children’s mothers during the earthquake and we did not assess the level of maternal stress during pregnancy neither in exposed nor unexposed children, so children could have been exposed to different stress levels, depending on how their mothers experienced and perceived the 27F earthquake and the aftershocks that followed this event. In this regard, a previous report identified that mothers exposed during pregnancy to the 27F earthquake and its immediate strong aftershocks experienced high levels of psychological distress^65^.

Another limitation is that we estimated the exposure timing based on the date of birth, which could have led to a potential misclassification of children, however, although distinct studies describe negative associations between prenatal stress and gestation length, in most of these reports the increase in preterm birth ratio was rather small^8,66–68^. Thus, even though it is likely that in the present study the mean gestational age of the exposed group was reduced, according to the literature, it is expected that the reduction was less than a week and thus it would not affect our classification of trimester-specific exposure and would not lead to a differential misclassification of the results^8,66–68^. Moreover, even though children exposed in the first trimester were likely exposed to all the effects of the earthquake, it seems that the mainshock and the aftershocks concentrated in the first two weeks after the mainshock (from February 27th to March 11st, 2010), were the most stressful events. Finally, the purposive sample might limit the external validity of our study, so the findings should be analyzed and interpreted considering this issue.

Despite these limitations, the fact that prenatal development is a critical period in the formation of most cognitive and non-cognitive skills, parents, educators, and policymakers should be attentive to negative shocks during in-utero period. Early interventions to remediate deficiencies might be more cost-effective than interventions at later ages; furthermore, early interventions might also contribute to alleviate some of the current social inequalities^64^. This work triggers some other questions and lines of work, such as: (i) the impact of prenatal exposure to 27F in children of different sociodemographic background, (ii) the association of children’s reading outcomes with prenatal exposure to other maternal stressors, and (iii) successful strategies to timely prevent or reduce the consequences of maternal distress during pregnancy.

## Methods

### Study design and setting

This multilevel retrospective cohort study analyzed secondary data collected from three cohorts of kindergarten children (five to six years old) who attended subsidized schools located in the Greater Santiago, Metropolitan Region, Chile (Supplementary Figure 1). The datasets contained pre-reading skills measured with the DIALECT platform, children’s sex (male/female), date of birth, classroom (A/B/C/D), and school for each cohort.

Due to the hierarchical structure of this study, we considered children as the first level, classrooms as the second level, and schools as the third level of analysis (Supplementary Figure 2).

### Data, variables, and sample

#### Outcome

Pre-reading skills were evaluated with the DIALECT platform, which is a validated, online, diagnostic Spanish reading assessment instrument that examines students’ performance in various reading sub-processes or reading precursors^47,48^. DIALECT tests are self-administered and untimed. Students listen to instructions for each subtest and mark their answers on a tablet. Content, construct, and concurrent validity for DIALECT^®^ have been reported in several studies and with large population samples^47,48^.

In this study, we analyzed five pre-reading skills: (i) listening comprehension (ability to understand spoken text); (ii) alphabet knowledge (ability to identify all the letters of the alphabet); (iii) print knowledge (ability to recognize several components and features of written text); (iv) phonological awareness (ability to discriminate and manipulate sounds in words), and (v) vocabulary (ability to understand the meaning of different words). Each skill was analyzed considering the total score obtained and the category of achievement associated with that score: category 1 (“delayed”), category 2 (“normal”), category 3 (“very good”), category 4 (“outstanding”), according to previous reports^49^ (Table 3).

DIALECT has been applied in more than 100 schools since 2013. In this particular study, we analyzed a purposive sample of 16 schools that belong to an educational network of 19 schools that serves children from low socioeconomic homes; therefore, social and demographic variables are likely to be homogeneous across all schools. All assessments were performed by kindergarten students at the beginning of each academic year (March) in 2015, 2016, and 2017.

#### Exposure

The exposure variable was prenatal stress, which was considered as the in-utero exposure to the 27F earthquake in 2010. The epicenter of this natural event was approximately 350 km southwest of the capital, Santiago. According to the United States Geological Survey, the earthquake had a magnitude of 8.8 on the Richter scale and duration of more than 3 min, the 27F earthquake became the fifth largest earthquake recorded to date^46^.

Children’s date of birth was used to estimate the timing of in-utero exposure. Children who were born before 27 February 2010 and after 10 December 2010 were considered to be unexposed to prenatal stress due to 27F. On the other hand, children who were born on 27 February 2010 and later but not beyond 10 December 2010, were considered exposed. Additionally, we analyzed trimester of exposure; thus, children were exposed during the first, second, or third trimester of gestation if they were born between 11 September and 10 December, 5 June and 10 September, 27 February and 4 June, respectively.

#### Covariates

Socioeconomic status might be a potential confounder, thus, we considered average municipality income as a proxy for this variable at the third level of analysis. Income data were based on the Agencia de Calidad de la Educación (Agency for Quality in Education) index of school vulnerability. In addition, within each school, each cohort were divided into two to four kindergarten classrooms, so they were included as the second level of analysis. Child’s sex was considered an effect modifier; therefore, it was included as a covariate at the first level of analysis.

### Ethics

Data were obtained and analyzed with the permission of schools’ director and this study was approved by the Universidad de Los Andes (Santiago, Chile) Ethics Committee (number of resolution CEC201933). All analyses were performed in accordance with the relevant guidelines and regulations of Chilean Legislation (described in laws number 20.120, 20.584 and 19.628 and in the legal normative from the Chilean Ministerial Advisory Commission for Health Research-CMEIS). The data were kept in a masked database and all analyses were conducted anonymously.

### Statistical analysis

Descriptive and bivariate analyses were conducted. The comparison of scores by exposure or trimester of exposure was performed using Mann–Whitney *U* test and Kruskal Wallis test, respectively. Complementary, the Dunn’s test was used to obtain pairwise comparisons among trimester of exposure. Chi^2^ test or Fisher’s exact test were used to evaluate the association between the category of achievement and exposure (unexposed versus exposed) or trimester of exposure.

Multilevel analyses considered three-levels, school-and-class as random effects, and were performed using multilevel generalised linear models for each outcome measured as categories of achievement (from delayed to outstanding). Exposure status (unexposed/exposed or trimester of exposure) and child’s sex (male/female) were considered predictor variables at the first level, whereas average municipality income was considered a predictor at school level. For modelling, we used ordinal family, logit link, estimated odds ratios (ORs) and considered p-values less than 0.05 to be statistically significant, except for Dunn’s tests (p-values less than 0.025 were considered statistically significant). All analyses were done in STATA IC version 15.

### Ethical approval

This study received ethical approval from the Institutional Review Board of the Universidad de Los Andes, Santiago, Chile (Number of resolution CEC201933). All analyses were conducted anonymously.

## Supplementary Information

Supplementary Figure 1.

Supplementary Figure 1.
