# Supplementary figures and images for: Maternal exposure to a high-magnitude earthquake during pregnancy influences pre-reading skills in early childhood
Source: Sci Rep. 2021 Apr 29;11:9244. doi: 10.1038/s41598-021-88767-7 (PMC8084950; doi:10.1038/s41598-021-88767-7)

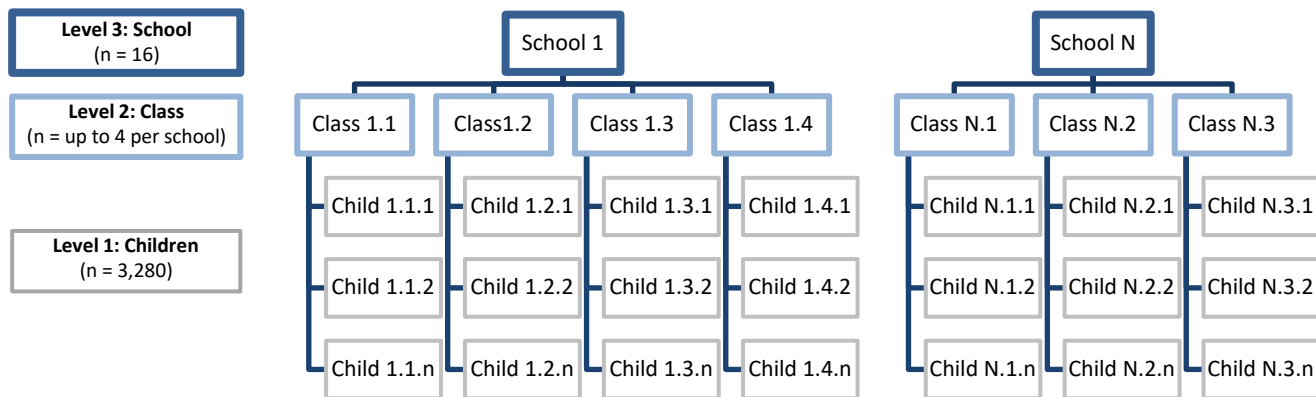

**Supplementary Figure 1.** Multilevel analysis diagram

Supplement: Supplementary file 1 — Supplementary Figure 1. [file 41598_2021_88767_MOESM1_ESM.pdf]

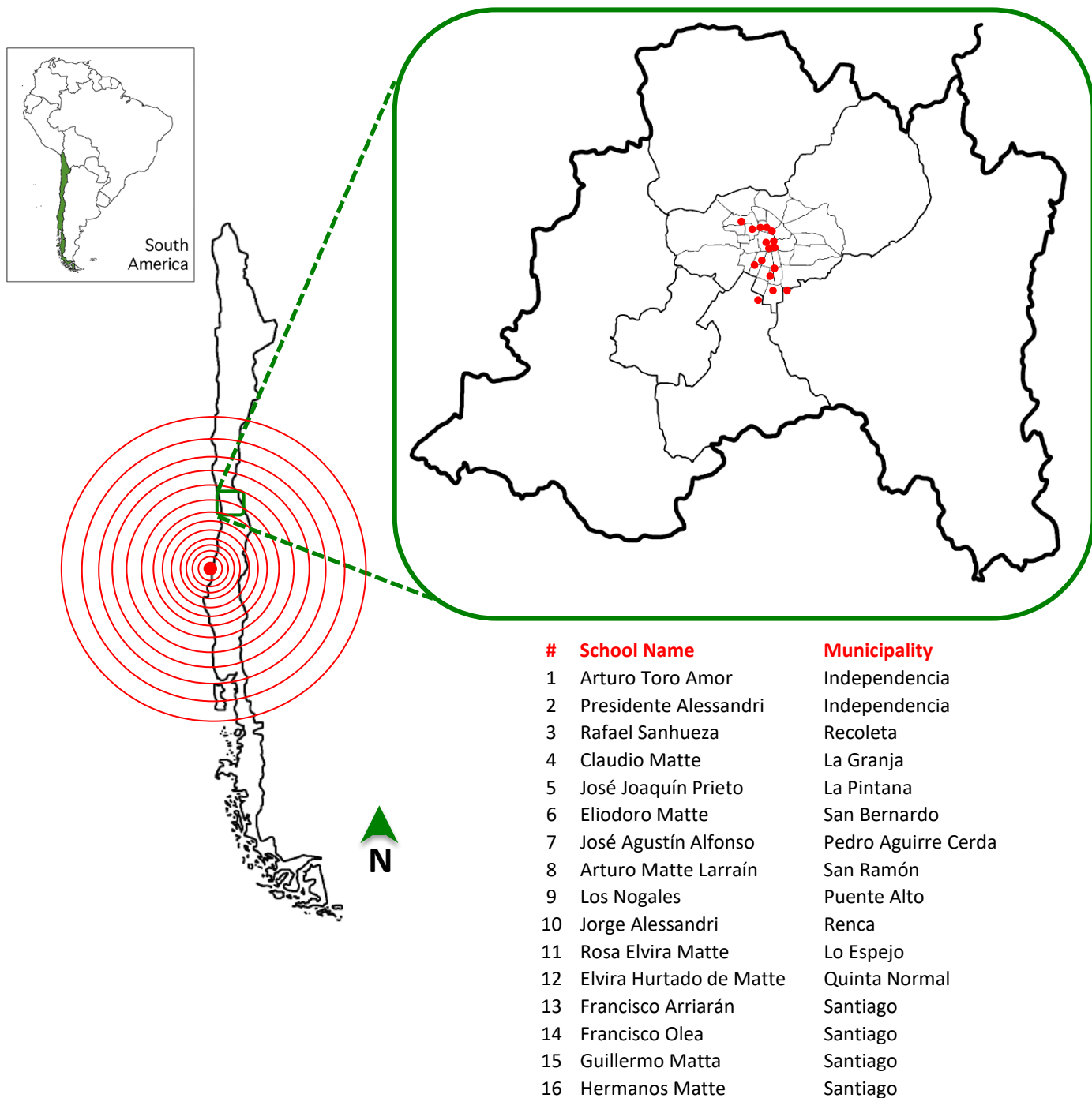

**Supplementary Figure 2.** Geographical location of schools included in this study (red dots).

Supplement: Supplementary file 2 — Supplementary Figure 1. [file 41598_2021_88767_MOESM2_ESM.pdf]
